# The Growing Need for Web-Based Simulation in Low- and Middle-Income Countries

**DOI:** 10.31729/jnma.8814

**Published:** 2024-11-30

**Authors:** Rakesh Ghimire, Sanjeev Kharel, Subarna Giri, Allan J. Hamilton

**Affiliations:** 1 Department of Clinical Pharmacology, Maharajgunj Medical Campus, Maharajgunj, Kathmandu, Nepal; 2 Maharajgunj Medical Campus, Maharajgunj, Kathmandu, Nepal; 3 Department of Neurosurgery, University of Arizona Health Sciences Center, USA

**Keywords:** *remote learning*, *simulation*, *telesimulation*, *virtual*, *virtual patients*

## Abstract

Simulation education is the bridge between learning clinical medicine in the classroom and delivering it at the bedside. As healthcare simulation has matured over the last two decades, it has begun to evolve many of the same methodologies. Rapid technological advancements across the fields of computer science, bioengineering, and curriculum design have helped to provide healthcare that is delivered more efficiently, effectively, and ethically. Web-based simulation programs (Web-SP) are poised to provide an efficient way to deliver asynchronous training in healthcare professionals' education. Web-SPs could also sponsor specialty-specific, web-based fellowships for clinicians of LMICs. The COVID-19 pandemic provided unique insight into the robustness of web-based learning tools that permitted remote learning opportunities. Under similar circumstances, should they arise again, Web-SPs would be a valuable tool for sustaining medical training under conditions where only remote learning may be feasible. Studies indicate that cost-effective simulation training can be delivered to learners in remote, low-resource areas worldwide, including South Asia, where access to such education is limited. We aimed to explore the effectiveness, challenges, and strategies for implementing web-based simulation education in low- and middle-income countries, based on a thorough PubMed search focused on web-based simulation programs in medical education.

## INTRODUCTION

Simulation is described as "the imitative representation of the functioning of one system or process through the operation of another."^1^ Healthcare simulation has matured dramatically in the last two decades and has begun to share much of the same established simulation methodologies that are ubiquitous within aviation, spaceflight, nuclear power, shipping, and the military. Advances in computer science, bioengineering, and simulation design are helping healthcare training that is safer, more effective, efficient, and ethical.^2^ There is growing evidence showing the benefits of incorporating simulation-based systems into medical education. Simulated patients (SPs; or even role-playing) have clear benefits even without using modern screen-based simulations. Virtual patients (VPs), on the other hand, allow users to learn at their desired pace, control the environment, and even use it to learn in a home setting.^3^ Similarly, web-based simulation programs (Web-SPs) can target the acquisition of both funds of knowledge as well as complex skill sets through the use of interactive and realistic VPs in medical education.^4^ The process of using simulation and telecommunication tools to give students instruction, training, and evaluation off-site is known as telesimulation.^5^ In procedural task training, telesimulation has been used in fields like robotic surgery, laparoscopic surgery, and intraosseous needle insertion.^6-8^ The objective of this narrative review is to explore the potential of Web-SPs in medical education mainly in low- and middle-income countries (LMICs) countries and to discuss its opportunities, challenges, and utilization in medical education. With this aim in mind, we searched databases such as PubMed, Embase, and Google Scholar using relevant keywords Web-Based Simulation, "Telesimulation," "Virtual Patients," and "Low and Middle-Income Countries." The findings from relevant articles are cited below.

## HISTORICAL BACKGROUND

In healthcare, using simulation to demonstrate, acquire, and maintain skills dates back a long history. Conventional apprenticeship was the norm for clinical education up to the 20^th^ century. ^9,10^ Early instructional tools and simulators ranged from straightforward casts or carvings to more complex materials that mimic human tissue in appearance, and texture and were utilized in an immersive and interactive way which we called simulation.^11^ Approximately 2500 years ago, according to Owen, surgical training program included simulation to develop skills needed by the medical students before their clinical practice. A national simulation-based training curriculum was created more than 300 years ago to instruct midwives, and these simulators could be configured to bleed and leaking amniotic fluid exactly like they do now. 9 Role-playing, standardized patients and part-task trainers developed in 1958 were the first step towards simulation.^12^ Laerdal's Resusci-Anne, Gaba's CASE 1.2, and Good and Gravenstein's GAS act as predecessors to the modern human-patient simulator.^11,13^

Stephen Abrahamson and Judson Denson introduced computer-patient interactions in 1966 with a highly developed manikin that could blink, breathe, have a heartbeat, pulse, blood pressure, move the mouth, and respond to medications.^14^ The latest SimMan 3G (Human Patient Simulator ) was introduced in 2009 and has paved the way for more advanced simulation programs.^12^ One of the most sophisticated emergency care patient simulators available is SimMan 3G PLUS, which is made for realistic simulations of scenarios. To improve learner competency, all aspects of medical instruction can be done simultaneously and with different degrees of difficulty in each scenario. Staff members can receive training in a risk-free setting through simulation. Frequent practice with intricate medical scenarios aids in the prevention of medical errors, and thorough feedback promotes dialogue and reinforces the learning process.^15^

## COVID-19 AND SIMULATION-BASED EDUCATION

After the declaration of a global pandemic in March 2020 by WHO 16 all aspects of medical education have had a significant impact by the pandemic. Since the beginning of the pandemic, approximately 2600 medical schools worldwide^17^ had to reorganize to sustain, remodel, and effectively deliver medical training during the crisis. Bentata described in detail how COVID-19 led to the suspension of medical school classes and/or hospital training rotations, instruction to be done entirely online, exams to be postponed, and delaying the commencement of a new academic year.^18^ According to a McGill University study, 74% of students said that since the pandemic started, the quality of their education has declined.^19^ According to 70% of trainees in a different trial by Ferrara et al., COVID-19 caused a 50% reduction in clinical activity and a 75% reduction in surgical activity.^20^ The COVID-19 pandemic has affected medical students not only negative ways but it has also had positive effects on a few medical students. SBT, or simulation-based training, has been used to fill up the gaps left by the pandemic and develop clinical skills. Dedeilia et al ^21^ described that medical education is resilient and adaptable in the face of problems like the worldwide pandemic of COVID-19 as medical student and resident patient engagement was restricted to cut down the transmission rate. During the COVID-19 pandemic, simulations have helped clinicians hone their use of personal protective equipment (PPE) and other infection control measures. Additionally, it has helped them in learning new and expanded roles, like processing equipment and assisting infected patients in prone position experiencing respiratory failure.^22^ Initially, telesimulation was utilized in resource-limited environments,^6-8^ it has been looked at as a reasonable alternative for replicating authentic patient experiences for medical students during the COVID-19 pandemic.^23^

## WEB-SP IN LMIC:

The efficacy of techniques like Script Concordance Testing, which have been approved by esteemed organizations like the American Heart Association and the European Resuscitation Council, points to their potential for application in residency programs and in training medical personnel for emergency circumstances.^24^ There is a demand for simulation-based medical education (SBME) in developing countries due to inadequate resources and faculty.^12^ A recent scoping review categorized the use of SBME in LMICs into two categories: medical education, training, and assessment, as well as the use of simulated patients. SBME is underutilized in LMICs across all medical subspecialties.^25^ There are few permanently established simulation-based training programs and centers where healthcare professionals and trainees can practice clinical skills. The simulation used to instruct and evaluate healthcare professionals and trainees in LMICs is also limited and erratic. Low funding further restricts the use of simulation in health professional education in LMICs.^26^

The use of simulation in medical subspecialties can be a unique and reasonable option in LMICs.^27^ The Center for Innovation in Medical Education at Aga Khan University has become the first simulation-based educational institution in South Asia to receive accreditation from the US Society for Simulation in Healthcare. It currently offers over 200 simulation-based courses ranging from basic life support to complex birth scenarios, all of which have helped healthcare professionals enhance their skills.^28^ There have been increased educational opportunities for learners with globalization, telesimulation, and widespread internet connectivity in remote locations. Which has broadened the scope and reach of simulation training. SPs may suffer costs associated with purchasing pricey equipment for on-site training, but they do not require a large capital expenditure.^6^ Web-SPs would permit simulation events in LMIC to be held regularly rather than sporadically.^25^

## SUCCESS

Web SPs have been commonly used because they allow optimal learning opportunities without the need to invest in expensive computerized mannequins. The financial burden of offering simulation training decreases because of the low initial cost of basic training materials needed, reduced need for travel costs, and reduced overhead for maintaining training space.^29^ Web SPs offer more flexible scheduling for simulation training. Simulated challenges can be tailored to the individual to approximate their level of practice better. People learn without worrying about the commission of errors and it helps build a level of experience and confidence before encountering real-life events.^30^ The introduction of web-enabled simulators in recent times will simplify the installation process for educational institutions, the accessibility of screen-based simulations for students, and the process of updating them with downloadable updates.^31^ A variety of potential novel avenues for screen-based simulation are also made possible by the internet, one of which is the ability to facilitate the management of a simulated scenario by several participants in a real-time, networked context. Screen-based simulation is expected to be a key component of future research to identify performance inadequacies that translate into opportunities for focused curriculum and care process improvement.^32^ Telesimulation has been utilized for instruction in critical care fields such as emergency medicine and pediatric anesthesia.^33,34^ Studies have shown that enhanced self-reported knowledge and confidence have been achieved through tele simulation which is in line with in-person simulation.^8,35,36^ A study done by Lin et al, demonstrated that the it is possible and well-received by students to implement a critical care case-focused simulation course using telesimulation.^23^ Telesimulation presents various benefits, such as the capacity to conduct training in settings with limited resources, the ability to evaluate learners from a distance, and the ability to get over geographical and temporal training barriers. This provides a unique opportunity for learners from LMICs.^37^

## DRAWBACK AND CHALLENGES

Technical errors in computer-based systems, and the cost of purchasing or upgrading simulation software are some disadvantages associated with using WB-SPs.^38^ Ensuring realistic, interactive, and authentic simulation is one of the challenges faced by the trainer.^39^

Significant exposure to real-world patient situations is necessary to develop and reinforce clinical reasoning. The amount of time that academic programs can expose students to actual patient cases is one of the limitations.^40^ To maximize student exposure, texts with illustrations and standardized patient experiences are typically utilized; nevertheless, there are drawbacks to each of these approaches. The lack of reproducibility, digital literacy, and user confidence are some drawbacks.^41^ The range of simulation modalities has also increased, moving from "high-fidelity" mannequin-based simulations to simulated patient approaches and finally to hybrid simulations that combine multiple simulation modalities.^42^

Many simulation centers have inadequate infrastructure support, poor research productivity, inadequate utilization of simulation technologies, and inadequate curricular integration. Due to these challenges, simulation in healthcare is unable to successfully branch out into other crucial domains like professional growth, patient safety, faculty development, and advocacy.^43^

The establishment of a link between distant medical educators from first-world countries with healthcare professionals in LMICs is needed for the possible collaboration between large centers. This would be the first step to creating an educational pipeline aimed at meeting the rising need to populate a web-based simulation network. A gradual deployment of qualified simulation educators along with a subsequent online "train the trainer" program would help to quickly establish a feasible and cost-effective program.^44^ Similarly, four factors are significant–learner requirements, educator vision, global collaboration, and sustainable funding in influencing the future direction and expansion of simulation worldwide.^43^ For long-term financial support, healthcare institutions need to formally include simulation in their budgets to include it into their curricula properly. At the same time, they should look at ways to collaborate with other regional centers along with improving patient safety.^43,45^ Effectiveness in simulation can be achieved through close supervision, mentoring, and practice-specific skills by the faculty in a controlled environment before displaying competency and proficiency. Furthermore, the skills checklist is a tool that can help learners learn even more in a simulated environment.^46^ These are the points that have to be taken into consideration for the effectiveness of the simulation program.

The use of web-based simulations in educational settings holds great promise for LMICs, offering opportunities to bridge learning gaps and improve educational outcomes. However, successfully implementing these simulations requires careful consideration of practical challenges unique to LMIC contexts.

There are numerous reasons to concentrate on developing virtual labs for university education. The expense and lack of a skill set necessary to handle the current boom in the biotechnology sector are two of the main causes. The expense of setting up laboratories also places a significant burden.^47^ Despite encouraging trial results, there are several obstacles this approach must overcome to be widely adopted. The multi-workshop format and mandatory supervision visits make this training technique more expensive per participant than other in-service courses. During supervisory visits, the results of some improvement projects could not be verified, and other projects were not continued. It takes time, desire, and steady support to change behavior.^48^ These projects do have certain constraints related to technological accessibility, a stable internet connection, user confidence, and digital literacy.^6,49^ The adaptability of SBME is another difficulty for LMICs.^47^ The pictorial diagram has also shown various challenges to SBME in LMICs.

**Figure 1:**
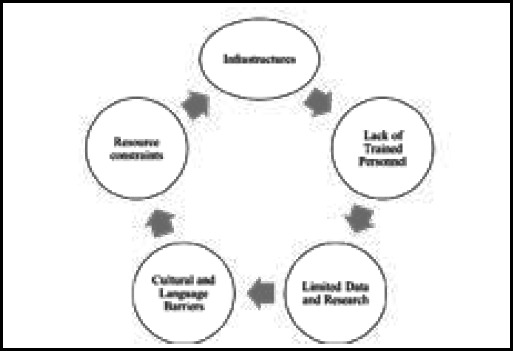
Pictorial framework showing challenges in LMIC.

## WAY FORWARD

Adapting to new technological advances is a human need. Studies focusing on the Web-SP in a low-resource setting are necessary. As each LMIC faces unique obstacles, experts and educators must devote focused effort to defining clear learning objectives, establishing effective feedback delivery, and determining which SBME mode to use.^46^ Technical problems that impact participation presented the biggest barrier during the sessions, as evidenced by studies under review that show learner satisfaction with telesimulation. Enhancing the quality of the audio and video also depended on things like internet access. Optimizing telesimulation learning thus requires sufficient technological preparation, simulation training, and the creation of evidence-based standards.^51^ The studies have shown the feasibility of providing cost-effective simulation training to learners in off-site locations, including remote and resource-limited areas worldwide that would otherwise lack access to this innovative educational approach. The success in Pakistan is an example to all South Asian Countries about the applicability and adaptability of Simulation in healthcare in low-resource settings mainly South Asian Countries.^29^ Similarly, its growing use in different residency and subspecialty programs puts it as an indispensable asset in medical education in low-resource settings. Telesimulation –a relatively new technique–draws parallels to telemedicine, another emerging field in medical education. Due to limited exposure to both strategies during training, many novice learners find it challenging to recognize their benefits in medical education. Participants must be aware of telesimulation basics, as goal creation and achievement may be hindered by a lack of familiarity with the approach.^54^ In an article by Thomas et al.,^12^ key tips for conducting medical telesimulation are outlined, including selecting case topics and materials that accommodate the limits of telesimulation compared to in-person simulation. While telesimulation allows learners to develop cognitive skills, such as clinical reasoning, and communication skills, including teamwork and history-taking, it does not facilitate practice of hands-on skills like airway management or procedural tasks. Consequently, cases that do not heavily depend on procedural performance are more suitable for this format.^55^ A national commitment is essential for the sustainable development of telesimulation in low- and middle-income countries (LMICs). Establishing a unified organization that includes relevant policy-making bodies and promotes cross-sectoral coordination is critical. Findings suggest that an effective approach to addressing challenges involves creating a well-defined roadmap through comprehensive feasibility studies and piloting telesimulation initiatives. Promoting community acceptance is also challenging in regions where traditional teaching methods are deeply rooted.^56^

**Table 1:** Comparative insights of challenges and its solutions of telestimulation in medicine according to geographical regions^5,6,25,46,48,50,52,53,54^

Regions	Challenges	Solutions
African	Serious financial constraints, poor infrastructure, and a lack of qualified teachers	International alliances and mobile simulation units
South Asia	Large student populations, unequal infrastructure development, and cultural resistance	Targeted teacher training and expandable simulation programs
South East Asia	Infrastructural variations and economic inequality	Shared resource centers and regional cooperation
Latin America	Financial limitations and administrative obstacles.	Public-private cooperation and policy advocacy.
Middle East	Political instability and regulatory variations	Regional stability and harmonizing regulations.
America	Funding variability, Regulatory and Accreditation Issues, Faculty training	Standardized Protocols, Grant funding, Faculty Developing Programs
Europe	Diverse economic condition, Language Barrier, Accreditation issues	European Union grants, Multilingual platforms, EU-wide Accreditation

## CONCLUSIONS

Web-SP is a way forward in LMIC despite the various challenges looking at the unmet needs. The use of Web SP offers opportunities for training in different medical and surgical specialties. Web-SP could be effective methods of risk-free environment learning and are particularly important to the healthcare professionals of resource-constraints settings in LMICs.
